# c-FLIP Degradation Mediates Sensitization of Pancreatic Cancer Cells to TRAIL-Induced Apoptosis by the Histone Deacetylase Inhibitor LBH589

**DOI:** 10.1371/journal.pone.0010376

**Published:** 2010-04-28

**Authors:** John Kauh, Songqing Fan, Mingjing Xia, Ping Yue, Lily Yang, Fadlo R. Khuri, Shi-Yong Sun

**Affiliations:** 1 Department of Hematology and Medical Oncology, Emory University School of Medicine and Winship Cancer Institute, Atlanta, Georgia, United States of America; 2 Department of Surgery, Emory University School of Medicine and Winship Cancer Institute, Atlanta, Georgia, United States of America; University of Hong Kong, Hong Kong

## Abstract

Great efforts have been made to develop novel and efficacious therapeutics against pancreatic cancer to improve the treatment outcomes. Tumor-necrosis factor-related apoptosis-inducing ligand (TRAIL) is such a therapeutic cytokine with selective killing effect toward malignant cells. However, some human pancreatic cancers are intrinsically resistant to TRAIL-mediated apoptosis or therapy. In this study, we have shown that the histone deacetylase inhibitor LBH589 can synergize with TRAIL to augment apoptosis even in TRAIL-resistant cells. LBH589 decreased c-FLIP levels in every tested cell line and survivin levels in some of the tested cell lines. Enforced expression of ectopic c-FLIP, but not survivin, abolished the cooperative induction of apoptosis by the combination of LBH589 and TRAIL, indicating that c-FLIP downregulation plays a critical role in LBH589 sensitization of pancreatic cancer cells to TRAIL. Moreover, LBH589 decreased c-FLIP stability and the presence of the proteasome inhibitor MG132 prevented c-FLIP from reduction by LBH589. Correspondingly, we detected increased levels of ubiqutinated c-FLIP in LBH589-treated cells. These data thus indicate that LBH589 promotes ubiqutin/proteasome-mediated degradation of c-FLIP, leading to downregulation of c-FLIP. Collectively, LBH589 induces c-FLIP degradation and accordingly sensitizes pancreatic cancer cells to TRAIL-induced apoptosis, highlighting a novel therapeutic regimen against pancreatic cancer.

## Introduction

Pancreatic cancer is one of the most difficult cancers to treat although it accounts for only 3% of all cancers. Despite multiple clinical trials with new chemotherapeutic agents, over the past 25 years the 5-year survival rate of 5%, and median survival of 6 months has largely remained unchanged. The median survival is about 6 months [1], [2]. One reason for the poor survival of pancreatic cancer is the insensitivity to most conventional therapies including chemotherapy and radiotherapy [3]. Thus, novel and efficacious therapeutic agents or regimens are urgently needed for treatment of pancreatic cancer.

Apoptosis is an essential part of mechanisms that maintain normal tissue homeostasis [4]. Deregulation of the apoptosis machinery and evasion of apoptosis is a general mechanism in cancer. Most chemotherapies act by the induction of apoptosis. Therefore, evasion of apoptosis is mainly responsible for the insufficiency of current therapies [2], [5]. It is well known that cells can die of apoptosis primarily through the extrinsic death receptor-induced pathway and/or the intrinsic mitochondria-mediated pathway [6]. The activation of the extrinsic death receptor-mediated apoptotic pathway involves ligation of a death ligand (e.g., tumor necrosis factor-related apoptosis-inducing ligand; TRAIL) with its corresponding cell surface death receptor(s) or aggregation (e.g. trimerization) of death receptors, leading to the formation of the death-inducing signaling complex (DISC) followed by the activating cleavage of caspase-8 in the DISC. Because Bid serves as a caspase-8 substrate, activation of the extrinsic death receptor apoptotic pathway also turns on the intrinsic apoptotic pathway [7].

The death ligand TRAIL has recently emerged as potential cancer therapeutic agent because it preferentially induces apoptosis in transformed or malignant cells [8]. Currently recombinant human TRAIL is being tested in phase I clinical trials. Moreover, agonistic antibodies against DR4 and DR5, which directly activate the extrinsic apoptotic pathway, have also been tested in phase I or II trials [9]. Thus, the death receptor, particularly the TRAIL death receptor mediated apoptosis has been under intense research as a cancer therapeutic target [10], [11]. Many preclinical studies have demonstrated therapeutic potential of targeting the TRAIL/death receptor-mediated apoptosis in pancreatic cancer [12]–[20]. However, an important issue in this regard is the intrinsic resistance of certain cancer cells including pancreatic cancer cells to TRAIL/death receptor-induced apoptosis [17], [18].

Cellular FLICE-inhibitory protein (c-FLIP), which inhibits caspase-8 activation by preventing recruitment of caspase-8 to DISC, is the primary inhibitor of TRAIL/death receptor-induced apoptosis [21], [22]. The levels of c-FLIP, including both FLIP_L_ and FLIP_S_ are subject to regulation by ubiquitin/proteasome-mediated degradation [23]–[25]. Elevated c-FLIP expression protects cells from death receptor-mediated apoptosis, whereas downregulation of c-FLIP by chemicals or small interfering RNA sensitizes cells to death receptor-mediated apoptosis [26]. Overexpression of c-FLIP has been suggested to be the key mechanism underlying TRAIL resistance in pancreatic cancer [13], [17].

LBH589 (panobinostat) is a pan-histone deacetylase (HDAC) inhibitor with promising anticancer activity [27]. Single-agent activity against pancreatic cancer has been demonstrated in preclinical experimental models [28]. In this study, we have revealed a novel activity of LBH589, which sensitizes pancreatic cancer cells to TRAIL-induced apoptosis. Moreover, we have shown that LBH589 facilitates ubiqutin/proteasome-mediated c-FLIP degradation, leading to enhancement of TRAIL-induced apoptosis in pancreatic cancer.

## Materials and Methods

### Reagents

LBH589 was provided by Novartis (Basel, Switzerland). The soluble recombinant human TRAIL was purchased from PeproTech, Inc. (Rocky Hill, NJ). The proteasome inhibitor MG132 and the protein synthesis inhibitor cyclohexemide (CHX) were purchased from Sigma Chemical Co. (St. Louis, MO). Rabbit polyclonal anti-DR5 antibody was purchased from ProSci Inc (Poway, CA). Mouse monoclonal anti-DR4 antibody (B-N28) was purchased from Diaclone (Stamford, CT). Mouse monoclonal anti-caspase-3 antibody was purchased from Imgenex (San Diego, CA). Rabbit polyclonal anti-XIAP, anti-caspase-8, anti-Mcl-1, and anti-PARP antibodies and mouse monoclonal anti-survivin antibody were purchased from Cell Signaling Technology, Inc. (Beverly, MA). Mouse anti Bcl-2 antibody was purchased from Santa Cruz Biotechnology, Inc (Santa Cruz, CA). Rabbit anti-GAPDH polyclonal antibody and mouse anti-Bax monoclonal antibody were purchased from Trevigen (Gaithersburg, MD). Mouse monoclonal anti-β-actin antibody was purchased from Sigma Chemical Co.

### Cell Lines and Cell Culture

Human pancreatic cancer cell lines used in this study were purchased from the American Type Culture Collection (Manassas, VA). For establishing pancreatic cancer cell lines that stably express ectopic c-FLIP or survivin, Panc-1 cells were infected with lentiviruses harboring lentiviral expression vectors of FLIP_L_ and survivin, respectively, as described previously [29], [30]. We also infected cells with lentiviruses carrying Lac Z expression vector as a control [29]. Individual cell clones resistant to blasticidin were expanded and subjected to screening of the expression of the targeted protein by Western blotting. These cell lines were cultured in DMSM medium containing 5% fetal bovine serum at 37°C in a humidified atmosphere of 5% CO_2_ and 95% air.

### Cell Survival Assay

Cells were seeded in 96-well cell culture plates and treated the next day with the agents indicated. The viable cell numbers were determined using the sulforhodamine B (SRB) assay, as previously described [31]. Combination index for drug interaction (e.g., synergy) was calculated using the CompuSyn software (ComboSyn, Inc.; Paramus, NJ). The statistical significance of differences between two treatments was analyzed with two-sided unpaired student's *t* tests by use of Graphpad InStat 3 software (GraphPad Software, San Diego, CA). Results were considered to be statistically significant at *P*<0.05.

### Detection of Apoptosis

Apoptosis was evaluated by annexin V staining using annexin V-PE apoptosis detection kit purchased from BD Biosciences (San Jose, CA) following the manufacturer's instructions. We also detected caspase activation by Western blotting (as described below) as an additional indicator of apoptosis.

### Western Blot Analysis

Whole-cell protein lysates were prepared and analyzed by Western blotting as described previously [32], [33].

### Immunoprecipitation for Detection of Ubiqutinated c-FLIP

Panc-1/FLIP_L_-5 cells, which stably express FLIP_L_, were transfected with HA-ubiquitin plasmid using the FuGENE 6 transfection reagent (Roche Diagnostics Corp., Indianapolis, IN) following the manufacturer's instruction. After 24 h, the cells were treated with LBH589 or MG132 plus LBH589 for 4 h and then were lysed for immunoprecipitation of Flag-FLIP_L_ using Flag M2 monoclonal antibody (Sigma Chemicals) as previously described [34] followed by the detection of ubiquitinated FLIP_L_ with Western blotting using anti-HA antibody (Abgent; San Diego, CA).

## Results

### LBH589 Sensitizes Pancreatic Cancer Cells to TRAIL-induced Apoptosis

**Figure 1:**
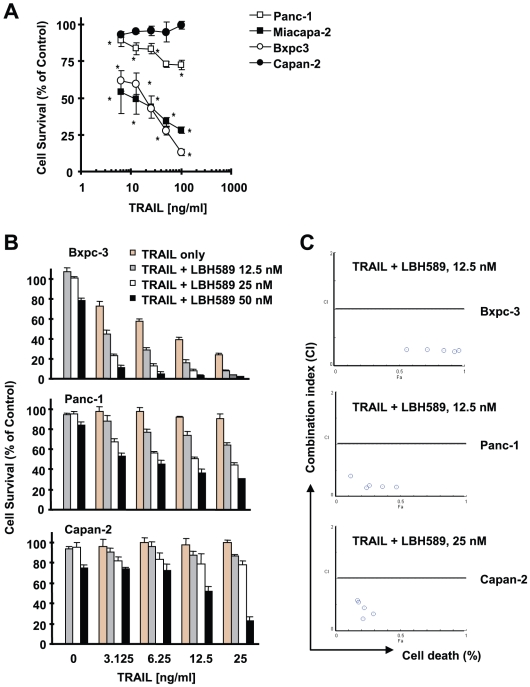
Responses of human pancreatic cancer cell lines to TRAIL (*A*) or to the combination of LBH589 and TRAIL (*B* and *C*). *A*, The indicated cell lines were seeded in 96-well cell culture plates and treated the next day with the given concentrations of TRAIL for 24 h. Cell numbers were estimated using the SRB assay. Data are the means of four replicate determinations; bars, ± SDs. *, *P*<0.01 compare with untreated cells. *B*, The indicated cell lines seeded in 96-well cell culture plates were treated with the given concentrations of TRAIL alone, LBH589 alone, or the respective combination of LBH and TRAIL for 24 h. Cell numbers were estimated using the SRB assay. Data are the means of four replicate determinations; bars, ± SDs. In Bxpc-3 and Panc-1 cells, each combination is significantly more effective than either TRAIL alone or LBH589 alone in decreasing cell survival (*P*<0.05 or <0.001). In Capan-2 cells, LBH589 at 50 nM in combination with 12.5 ng/ml or 25 ng/ml is significantly more effectively than LBH589 or TRAIL alone in decreasing cell survival (*P*<0.001). So are the LBH589 at 25 nM or 50 nM combined with TRAIL (*P*<0.05). *C*, Combinations indexes (CIs) were calculated based on the data presented in Fig. 1B using CompuSyn software.

**Figure 2:**
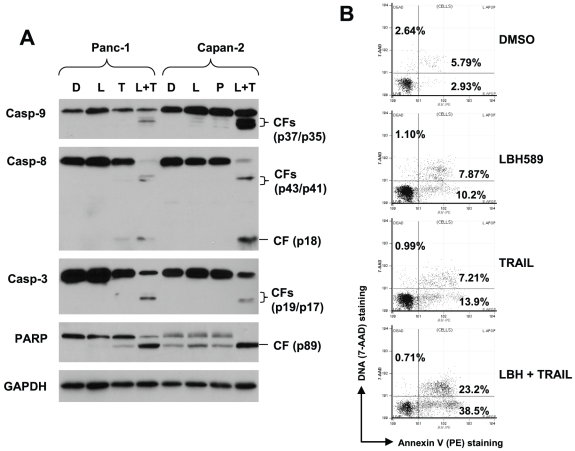
The LBH589 and TRAIL combination augments caspase activation (*A*) and apoptosis (*B*). *A*, The indicated cell lines were treated with DMSO control, 50 nM LBH589 25 ng/ml TRAIL alone, or LBH589 plus TRAIL. After 16 h, the cells were subjected to preparation of whole-cell protein lysates for detecting caspase cleavage using Western blotting. Casp, caspase; CF, cleaved fragment. *B*, Panc-1 cells were treated with 25 ng/ml TRAIL alone, 50 nM LBH589 alone or their combination for 24 h. The cells were then subjected to measurement of apoptosis using annexin V staining. The percent positive cells in the upper right and lower right quadrants represent the total apoptotic cell population.

We first determined the sensitivities of pancreatic cancer cell lines used in this study to TRAIL. As presented in Fig. 1A, four pancreatic cancer cell lines showed differential sensitivities: MiaPaCa-2 and Bxpc3 exhibited dose-dependent decrease in cell survival upon TRAIL treatment and thus were sensitive to TRAIL, whereas Panc-1 and Capan-2 were resistant to TRAIL because they showed minimal response to TRAIL in terms of decrease in cell survival. When combined with LBH589, enhanced cell-killing effects were observed not only in TRAIL-sensitive cells (e.g., Bcpc-3), but also in TRAIL-resistant cell lines (e.g., Panc-1 and Capan-2) because the combination of LBH589 and TRAIL were much more than either agent alone in decreasing the survival of the pancreatic cancer cells (Fig. 1B). The combination indexes for LBH589 (e.g., 12.5 nM) and TRAIL (3.125–26 ng/ml) combination in the tested cell lines were <0.5 (Fig. 1C), indicating that LBH589 and TRAIL combination exerts synergistic effects on decreasing cell survival of pancreatic cancer cells. Moreover, we directly detected apoptosis by measuring annexin V-positive cells and caspase cleavage in cells exposed to LBH589 alone, TRAIL alone and their combination. In agreement with cell survival data, the combination of LBH589 and TRAIL was much more potent than each single agent alone in inducing cleavage of caspase-9, caspase-8, caspase-3 and PARP (Fig. 2A) and increasing annexin V-positive cells (i.e., apoptotic cells) (Fig. 2B). Specifically, LBH589 and TRAIL alone caused approximately 18% and 21% apoptosis, respectively; however, the combination of LBH589 and TRAIL induced about 62% apoptosis, which is obviously greater than additive effect. Collectively, these results indicate that LBH589 sensitize pancreatic cancer cells to TRAIL-induced apoptosis.

### LBH589 Decreases the Levels of c-FLIP and Survivin in Pancreatic Cancer Cells

**Figure 3:**
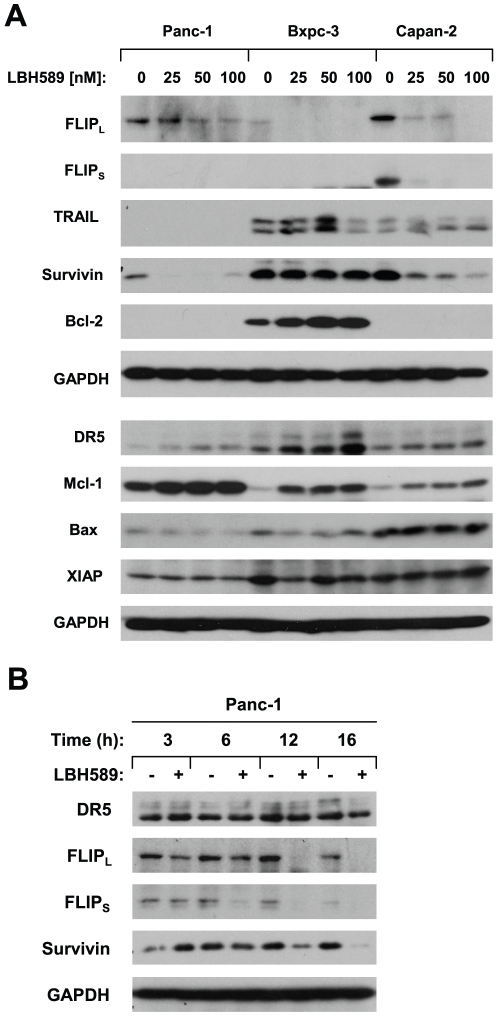
LBH589 modulates the levels of c-FLIP, survivin and other apoptosis-related proteins. The given cell lines were treated with different concentrations of LBH589 as indicated for 12 h (*A*) or with 50 nM LBH589 for the indicated times (*B*). After the treatments, the cell lines were subjected to preparation of whole-cell protein lysates and subsequent Western blot analysis for detection of the indicated proteins.

To understand the mechanisms by which LBH589 sensitizes pancreatic cancer cell lines to TRAIL-induced apoptosis, we first analyzed the modulatory effects of LBH589 on c-FLIP, DR5, DR4 and TRAIL, which are directly involved in regulation of the TRAIL/death receptor-mediated apoptosis, in three pancreatic cancer cell lines. Panc-1 and Capan-2 cells had higher basal levels of c-FLIP (particularly FLIP_L_) than Bxpc-3 cells. Treatment of these cell lines with LBH589 decreased the levels of c-FLIP in all of the three cell lines in a concentration-dependent manner (Fig. 3A). The c-FLIP reduction occurred at 3 h and became even more pronounced at 12 h post and thereafter post LBH589 treatment (Fig. 3B). LBH589 did not alter the levels of TRAIL in either of the tested cell lines (Fig. 3A) and only minimally increased DR5 expression in one of the three tested cell lines (i.e., Bxpc-3) (Figs. 3A and 3B). These results clearly suggest that c-FLIP donwregulation is an important event induced by LBH589.

In addition, we also examined the modulatory effects of LBH589 on other proteins including survivin, XIAP, Bcl-2, Mcl-1, and Bax, which regulate the mitochondria-mediated apoptosis. LBH589 decreased survivin levels in Panc-1 and Capan-2 cells, but not Bxpc-3 cells (Fig. 3A). Time course analysis of survivin levels in Panc-1 cells demonstrated that the pronounced survivin reduction occurred at 12 h post LBH589 treatment (Fig. 3B). LBH589 did not alter the levels of Bax and XIAP in these cell lines; however, it increased the levels of Mcl-2 in these cell lines as well as Bcl-2 levels in Bxpc-3 cells (Fig. 2A). Together, these results also suggest that survivin reduction may also be an important event induced by LBH589.

### Enforced Expression of Ectopic c-FLIP, but not Survivin, Protects Cells from Induction of Apoptosis by the Combination of LBH569 and TRAIL

**Figure 4:**
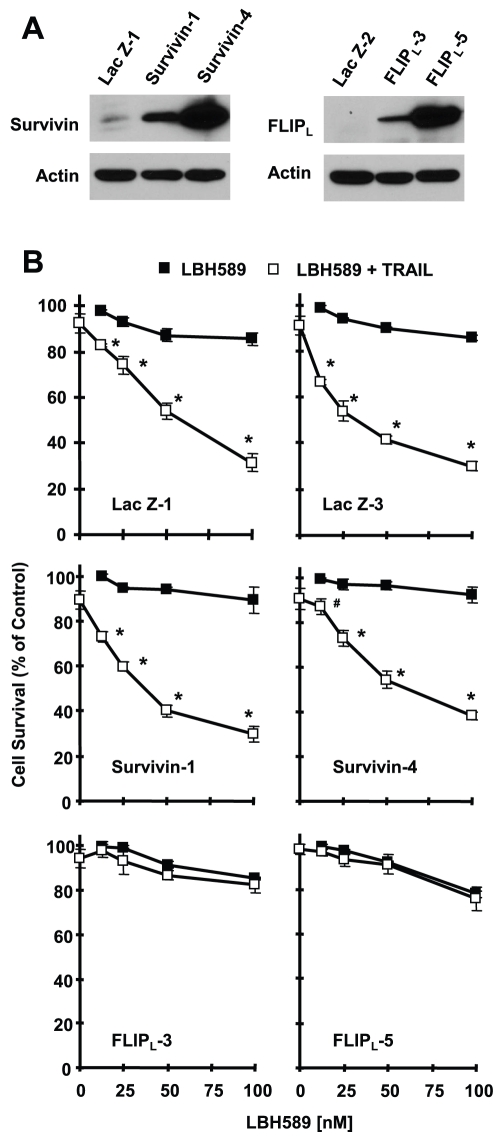
Enforced expression of ectopic c-FLIP, but not survivin (A), confers cell resistance to cell-killing by LBH589 and TRAIL combination (B). *A*, The expression of ectopic survivin or c-FLIP in the various trasnfectants as indicated was detected by Western blotting with survivin or c-FLIP antibody. *B*, The given transfectants were seeded in 96-well plates and treated with the indicated concentrations of LBH589 alone, 25 ng/ml TRAIL alone, or individual combination of LBH589 with TRAIL. After 24 h, the cells were subjected to the SRB assay for measurement of cell survival. Data are the means of four replicate determinations; bars; ± SDs. *, *P*<0.0001 and #, *P*<0.001 compared with LBH589 treatment alone.

**Figure 5:**
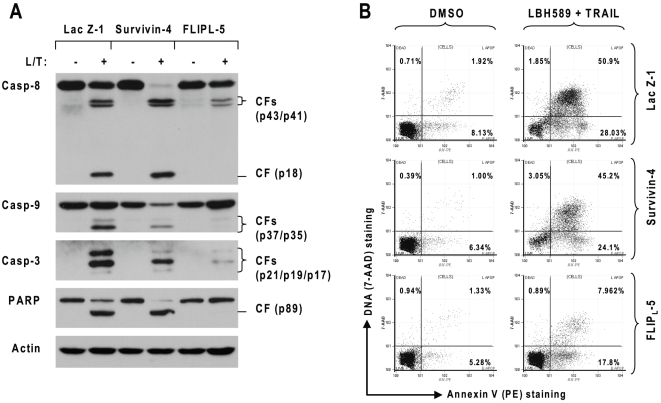
Enforced expression of ectopic c-FLIP, but not survivin, confers cell resistance to caspase activation (A) and apoptosis (B) induced by the LBH589 and TRAIL combination. The indicated transfectants were treated without and with 50 nM LBH589 plus 25 ng/ml TRAIL (L/T) for 16 h (*A*) or 24 h (*B*). The cells were then harvested for preparation of whole-cell protein lysates for detecting caspase and PARP cleavage using Western blotting (*A*) or for measurement of apoptosis using annexin V staining (*B*). The percent positive cells in the upper right and lower right quadrants represent the total apoptotic cell population.

Both c-FLIP and survivin are involved in regulation of TRAIL cell sensitivity [35]. To determine the involvement of c-FLIP and survivin downregulation in sensitization of pancreatic cancer cells to TRAIL-induced apoptosis by LBH589, we established Panc-1 cell lines that stably expressed ectopic FLP_L_ or survivin through a lentivial expression system and then examined their responses to the combination of LBH589 and TRAIL. The expression of ectopic survivin or c-FLIP was assumed by Western blotting as presented in Fig. 4A. Lac Z is an irrelevant protein and here was used as a control. As demonstrated above, the combination of LBH589 and TRAIL effectively decreased cell survival in Lac Z- or survivin-expressing cell lines, but failed to do so in both cell lines that express ectopic FLIP_L_ (Fig. 4B), indicating the enforced expression of ectopic FLIPL, rather than survivin, confers cell resistance to augmented induction of apoptosis by LBH589 and TRAIL combination. By detecting apoptosis, we found that the combination of LBH589 strongly induced cleavage of caspase-8, caspase-9, caspase-3 and PARP in panc-1 cell lines that express Lac Z or survivin, but only minimally in FLIP_L_-expressing Panc-1 cells (Fig. 5A). In agreement, the combination of LBH589 and TRAIL caused approximately 79% and 69% of apoptosis in Panc-1/lac Z-1 and Panc-1/survivin-4 cells, respectively, but only about 25% of apoptosis in Panc-1/FLIPL-5 cells (Fig. 5B), further confirming that FLIP_L_ overexpression confers cell resistance to the combination of LBH589 and TRAIL. Collectively, these results demonstrate that c-FLIP downregulation plays a key role in LBH-589-mediated sensitization of pancreatic cancer cells to TRAIL-induced apoptosis.

### LBH589 Donwregulates c-FLIP through Promoting Ubiqitin/proteasome-mediated Degradation

**Figure 6:**
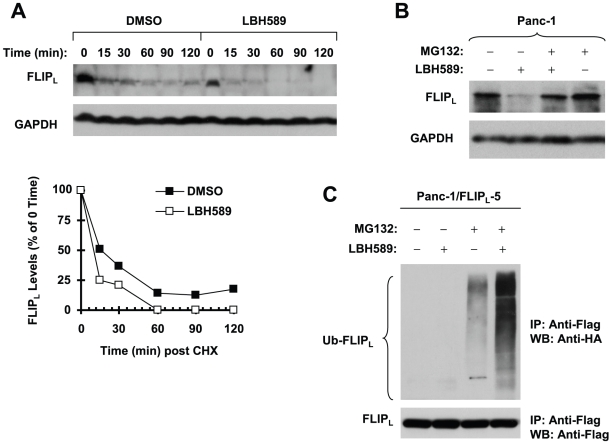
LBH589 reduces c-FLIP levels through ubiquitin/proteasome-mediated protein degradation. *A*, Panc-1 cells were treated with DMSO or 50 nM LBH589 for 4 h. The cells were then washed with PBS 3 times and refed with fresh medium containing 10 µg/ml CHX. At the indicated times post CHX, the cells were harvested for preparation of whole-cell protein lysates and subsequent Western blot analysis. Protein levels were quantitated with NIH Image J software (Bethesda, MA) and were normalized to GAPGH. The results were plotted as the relative c-FLIP levels compared to those at the time 0 of CHX treatment (lower panel). *B*, Panc-1 cells were pretreated with 20 µM MG132 for 30 minutes prior to the addition of 50 nM LBH589. After co-treatment for 4 h, the cells were harvested for preparation of whole-cell protein lysates and subsequent Western blot analysis. *C*, Panc-1/FLIP_L_-5 cells which stably express ectopic flag-FLIP_L_ were transfected with HA-ubiquitin plasmid using FuGENE 6 transfection reagent for 24 h. The cells were then pretreated with 20 µM MG132 for 30 minutes and then co-treated with 50 nM LBH589 for 4 h. Whole-cell protein lysates were then prepared for immunoprecipitation (IP) using anti-Flag antibody followed by Western blotting (WB) using anti-HA antibody for detection of ubiquitinated FLIP_L_ (Ub-FLIP_L_) and anti-Flag antibody for detection of ectopic FLIP_L_.

Given the critical role of c-FLIP downregulation in mediating enhancement of TRAIL-induced apoptosis by LBH589 as demonstrated above, we further addressed how LBH589 decreased c-FLIP levels. Because c-FLIP proteins are known to be regulated by ubiquitin/proteasome-mediated degradation [23], [25], we then determined whether the observed downregulation of c-FLIP by LBH589 would be mediated via this process. Thus, we first examined whether LBH589 promotes c-FLIP degradation. To this end, we treated Panc-1cells with either DMSO or LBH589 for 4 h and then washed away the drug followed by refilling the cells with fresh medium containing the protein synthesis inhibitor CHX. At the indicated times post CHX, the cells were harvested for Western blotting to analyze c-FLIP degradation rate. As presented in Fig. 6A, the reduction or degradation rate of FLIP_L_ in LBH589-treated cells was apparently faster than that in DMSO-treated control cells, indicating that LBH589 indeed facilitates c-FLIP degradation. Next, we treated cells with LBH589 in the absence and presence of the proteasome inhibitor MG132 and then compared c-FLIP modulation under these conditions. As presented in Fig. 6B, LBH589 decreased c-FLIP levels in the absence of MG132, but not in the presence of MG132, suggesting that LBH589-induced c-FLIP degradation is proteasome-dependent. By immunoprecipitation/Western blotting, we also detected the highest levels of ubiqutinated FLIP_L_ in cells treated with LBH589 plus MG132 compared to cells exposed to LBH589 alone or MG132 alone (Fig. 6C), indicating that HNK increases c-FLIP ubiquitination. Taken together, we conclude that LBH589 induces ubiquitin/proteasome-mediated c-FLIP degradation, leading to downregulation of c-FLIP in human pancreatic cancer cells.

## Discussion

Human pancreatic cancer tumors or cell lines exhibit heterogeneous responses to TRAIL. Some of these tumors or cell lines are intrinsically insensitive to TRAIL-induced apoptosis [17], [18]. In this study, we have presented a novel finding that the histone deacetylase inhibitor LBH589 effectively augments TRAIL-induced apoptosis in human pancreatic cancer cells including those resistant to TRAIL-induced apoptosis. Given that LBH589 shows anticancer activity in preclinical pancreatic cancer models [28] as well that the tumor-selective TRAIL is a potential cancer therapeutic protein and is being tested in phase I clinical trials, our findings warrant further evaluation on the combination of LBH589 and TRAIL as a potential therapeutic regimens against pancreatic cancer in animal models and in clinical trials.

Both survivin and XIAP are suggested to regulate TRAIL-mediated apoptosis [12], [36], [37]. Some HDAC inhibitors such as sodium butyrate and LAQ824 were reported to augment TRAIL-induced apoptosis involving donwregualtion of survivin and XIAP [38], [39]. A recent study has suggested that LBH589 enhances TRAIL-induced apoptosis through downregulation of XIAP in mesothelioma cells [40]. In our study, we found that LBH589 decreased survivin levels in two (i.e., Panc-1 and Capan-2) of three tested pancreatic cancer cell lines but did not obviously alter the levels of XIAP (Fig. 3). Moreover, enforced expression of ectopic survivin did not confer resistance to LBH589/TRAIL-induced apoptosis (Figs. 4 and 5). Thus, survivin and XIAP are unlikely to be involved in regulation of LBH589-mediated sensitization of TRAIL-induced apoptosis in pancreatic cancer cells.

Bcl-2 family members such as Bcl-2 and Mcl-1 have also been suggested in regulation of TRAIL-induced apoptosis [14], [35]. Other HDAC inhibitors enhance TRAIL-induced apoptosis in different cancer cells involving modulation of Bcl-2 family members such as downregulation of Bcl-2 and Bcl-X_L_ and upregulation of Bax and Bim [39], [41]–[44]. In our study, LBH589 did not change Bax levels. Unexpectedly, LBH589 increased the levels of Bcl-2 and Mcl-1 (Fig. 3). Thus, the modulation of these proteins is unlikely to be associated with LBH589-mediated potentiation of TRAIL-induced apoptosis in these cell lines; rather, increase in Bcl-2 and Mcl-1 may counteract LBH589's effect in sensitizing pancreatic cancer cells to TRAIL-induced apoptosis. Thus, further inclusion of a Bcl-2 or Mcl-1 inhibitor to this regimen may result in even more efficacious anticancer efficacy than the combination of LBH589 and TRAIL and should be further explored.

DR5 induction and c-FLIP downregulation are important mechanisms underlying drug-mediated augmentation or sensitization of TRAIL-induced apoptosis [45]. In our study, we found that LBH589 either did not or only weakly increased DR5 expression in pancreatic cancer cell lines (Fig. 3), suggesting that DR5 modulation has a limited role in LBH589-mediated sensitization of TRAIL-induced apoptosis in these cells. c-FLIP levels have been suggested to be associated with the sensitivity of pancreatic cancer cells to TRAIL-induced apoptosis; specifically, higher levels of c-FLIP was detected in the TRAIL-resistant pancreatic cancer cell lines compared with the TRAIL sensitive cells [17]. Inhibition of c-FLIP with a small interfering RNA or a small molecule sensitizes pancreatic cancer cells to TRAIL-induced apoptosis [13], [17]. Moreover, other HDAC inhibitors such as LAQ824, MS-275, FR901228, valproic acid and droxinostat have been shown to downregulate c-FLIP levels and enhance death receptor-induced apoptosis [46]–[52]. In our study, we also found that the TRAIL-resistant cell lines Panc-1 and Capan-2 had higher basal levels of c-FLIP than the TRAIL-sensitive cell line (Bxpc-3) (Fig. 3A). Like other HDAC inhibitors, LBH589 decreased c-FLIP cell lines in these three tested cell lines; this c-FLIP downregulation is a rapid event because c-FLIP reduction was detected even at 3 h post LBH589 treatment (Fig. 3). Importantly, enforced expression of ectopic c-FLIP (i.e., FLIP_L_) abolished LBH589's ability to enhance TRAIL-induced apoptosis (Figs. 4 and 5). Collectively, these results indicate that downregulation of c-FLIP is critical for LBH589-mediated sensitization of pancreatic cancer cells to TRAIL-induced apoptosis.

c-FLIP is known to be regulated by ubiquitin/proteasome-mediated degradation [23], [24]. Previous studies have shown that c-FLIP downregulation induced by certain HDAC inhibitors occurs at mRNA level [39], [51]. How HDAC inhibitors downregulate c-FLIP levels has not been fully elucidated. In our study, we found that LBH589 facilitated c-FLIP degradation as demonstrated in CHX chase assay. The presence of the proteasome inhibitor MG132 prevented c-FLIP from reduction induced by LBH589. Moreover, LBH589 increased the levels of ubiquitinated c-FLIP (Fig. 6). Thus, these results indicate that LBH589 facilitates ubqitin/proteasome-mediated c-FLIP degradation, resulting in c-FLIP downregulation. To the best of our knowledge, the finding on c-FLIP degradation or downregulation by LBH589 is novel and warrants further investigation on how inhibition of histone deacetylase leads to c-FLIP degradation.

The maximal plasma concentrations of LBH589 in human cancer patients range from 200 nM to 1300 nM depending on doses tested [53]. The concentrations of LBH589 used in our study that downregulate c-FLIP and enhance TRAIL-induced apoptosis are between 12.5 nM and 100 nM and thus within clinically achievable range. Therefore, the future clinical test of the combination is warranted.
